# Cellular and Molecular Mechanisms of Pristimerin in Cancer Therapy: Recent Advances

**DOI:** 10.3389/fonc.2021.671548

**Published:** 2021-05-07

**Authors:** Run-Ze Chen, Fei Yang, Min Zhang, Zhi-Gang Sun, Nan Zhang

**Affiliations:** ^1^ Jinan Central Hospital, Cheeloo College of Medicine, Shandong University, Jinan, China; ^2^ Department of Pathology, Jinan Central Hospital, Cheeloo College of Medicine, Shandong University, Jinan, China; ^3^ Department of Dermatology, Jinan Central Hospital, Cheeloo College of Medicine, Shandong University, Jinan, China; ^4^ Department of Thoracic Surgery, Jinan Central Hospital, Cheeloo College of Medicine, Shandong University, Jinan, China; ^5^ Department of Oncology, Jinan Central Hospital, Cheeloo College of Medicine, Shandong University, Jinan, China

**Keywords:** apoptosis, cancer, metastasis, molecular mechanisms, pristimerin, traditional Chinese medicine

## Abstract

Seeking an efficient and safe approach to eliminate tumors is a common goal of medical fields. Over these years, traditional Chinese medicine has attracted growing attention in cancer treatment due to its long history. Pristimerin is a naturally occurring quinone methide triterpenoid used in traditional Chinese medicine to treat various cancers. Recent studies have identified alterations in cellular events and molecular signaling targets of cancer cells under pristimerin treatment. Pristimerin induces cell cycle arrest, apoptosis, and autophagy to exhibit anti-proliferation effects against tumors. Pristimerin also inhibits the invasion, migration, and metastasis of tumor cells *via* affecting cell adhesion, cytoskeleton, epithelial-mesenchymal transition, cancer stem cells, and angiogenesis. Molecular factors and pathways are associated with the anti-cancer activities of pristimerin. Furthermore, pristimerin reverses multidrug resistance of cancer cells and exerts synergizing effects with other chemotherapeutic drugs. This review aims to discuss the anti-cancer potentials of pristimerin, emphasizing multi-targeted biological and molecular regulations in cancers. Further investigations and clinical trials are warranted to understand the advantages and disadvantages of pristimerin treatment much better.

## Introduction

Cancer is a severe health problem worldwide, which has posed a threat to the public for a long time. Myriads of therapeutic approaches have been applied in cancer treatment, such as chemotherapy, radiotherapy, immunotherapy, genetic and targeted therapy, hormonal therapy, and surgery, among which chemotherapy remains one of the most effective options for tumors. However, the application of chemotherapy can be limited by multidrug resistance (MDR) in cancer cells and severe side effects of drugs (1, 2). Thus, developing more effective therapeutic strategies is necessary. Traditional Chinese medicine (TCM) has been practiced for thousands of years, becoming a promising way for cancer therapy due to its minimal side effects and high efficiency (3, 4). Amounting evidence has shown that many natural products originated from traditional Chinese medicine exert effects against cancers such as resveratrol (5), dioscin (6), berberine (7), curcumin (8), and pristimerin (9).

Pristimerin, a naturally occurring quinone methide triterpenoid, belongs to the Celastraceae and Hippocrateaceae families. Bhatnagar and Divekar first extracted pristimerin from Pristimerae indica and P.grahami in 1951 and confirmed its structure in 1954 (10). Since then, more and more researchers have focused more on the therapeutic benefits of pristimerin and gained many advances. It has been reported that pristimerin exhibits many biological effects, such as insecticidal (11), anti-inflammatory (12), anti-angiogenic (13), anti-bacterial (14), anti-viral (15), anti-fungal (16) and anti-tumor effects (9). Pristimerin exhibits pharmacological effects against tumors through different targets and signal transduction pathways, including sonic hedgehog (Shh)/glioma-associated oncogene homolog 1 (Gli1) (17), phosphatidylinositol 3-kinase (PI3K)/Akt (18), erythropoietin-producing hepatoma receptor B4 (EphB4)/CDC42/neural Wiskott-Aldrich syndrome protein (N-WASP) (19), nuclear factor-kappaB (NF-κB) (20), reactive oxygen species (ROS)/mitogen-activated protein kinase (MAPK) (21), Wnt/β-catenin (22), insulin-like growth factor type 1 receptor (IGF-1R) (23), and hypoxia inducible factor 1α (HIF-1α)/sphingosine kinase 1 (SPHK1) (24)pathways as well as micro RNA (9), proteasome (25) and telomerase (26). Pristimerin suppresses cancer cell proliferation *via* G1 phase arrest (27), apoptosis (28) and autophagy induction (21). It also inhibits metastatic abilities of tumor cells through regulating cell adhesion (29), cancer stem cells (CSCs) (30), epithelial-mesenchymal transition (EMT) (31) and angiogenesis (13). The chemoresistance that occurred in cancer cells can be reversed under pristimerin treatment (32). Accumulating studies have also identified synergistic effects between pristimerin and other chemo-drugs (33, 34). The anti-tumor effects of pristimerin have been confirmed in various cancers, such as breast cancer (9), prostate cancer (24), and colorectal cancer (CRC) (6). This review aims to provide a timely overview of recent researches regarding anti-cancer activities and critical mechanisms of pristimerin against malignancies, providing potential directions and developments for future studies.

## Alterations in Essential Cellular Events Under Pristimerin Treatment

### G1 Phase Arrest Induction

There are four successive phases in the cell cycle, including G1 phase, S phase, G2 phase, and M phase. DNA synthesis occurs in S phase and nuclear division occurs in M phase (35). G1 phase is the gap between M and S phases, determining whether the cell can enter the S phase through G1 phase. G2 phase is incorporated after S phase, allowing the cell to prepare for mitosis (36). If the cell fails to cross the restriction point in G1 phase, it will stagnate at a quiescent state called G0 phase. Tumor is characterized by uncontrolled cell proliferation, which can be triggered by dysregulated cell cycle proteins. Thus, targeting cell cycle proteins has become a promising strategy for cancer treatment (37).

Emerging evidence has shown that pristimerin treatment gives rise to cell accumulation in G0/G1 phases and cell reduction in S and G2/M phases, indicating the induction of G0/G1 phase arrest to exhibit anti-proliferative effects on various cancers including CRC (22, 38), breast cancer (9, 21), uveal melanoma (UM) (18, 23, 30), chronic myelogenous leukemia (CML) (39), oral squamous cell carcinoma (OSCC) (27), esophageal cancer (40), pancreatic cancer (26, 41), prostate cancer (24, 25) and cholangiocarcinoma (42). However, a study related to the imatinib-resistant CML has found that pristimerin does not significantly affect the cell cycle other than the emergence of cells in the sub-G1 apoptotic phase (43). Cyclin D-CDK4/6 and cyclin E-CDK2 command a central position in the G1 progression through the restriction point (44). Pristimerin has been reported to downregulate the levels of cyclinD1 and CDK4/6 in breast cancer cells (21), CML cells (39), and colorectal cancer (CRC) cells (38). The decreased expression of cyclinD1 and cyclinE has also been observed accompanied by the reduction of CDK2/4/6 in prostate cancer cells (25), and pancreatic cancer cells (41). It has been revealed that the inhibition of CDK4/6 is closely associated with the inhibition of retinoblastoma (Rb) protein phosphorylation (45). Consistent with this demonstration, Yousef et al. have found that pristimerin attenuates the phosphorylation of Rb without dramatic changes in total Rb protein levels in CRC cells (38). Activation of the p53 tumor suppressor leads to the transient expression of the cyclin-dependent kinase inhibitor (CKI) p21, consequently results in not only G1 cell cycle arrest but also a chronic state of senescence or apoptosis (46, 47). Furthermore, p27, another cyclin-dependent kinase inhibitor, can bind to just like p21 to suppress its catalytic activity and induce cell cycle arrest (48). The elevation of p53, p21 and p27 levels under pristimerin treatment has also been found in breast cancer (21), OSCC (27), and pancreatic cancer (41). Therefore, it can be concluded that pristimerin induces G1 phase arrest in tumor cells *via* inhibiting the expression of cyclinD1/E and CDK2/4/6, while stimulating the expression of p53, p21 and p27.

### Apoptosis Induction

**Figure 1:**
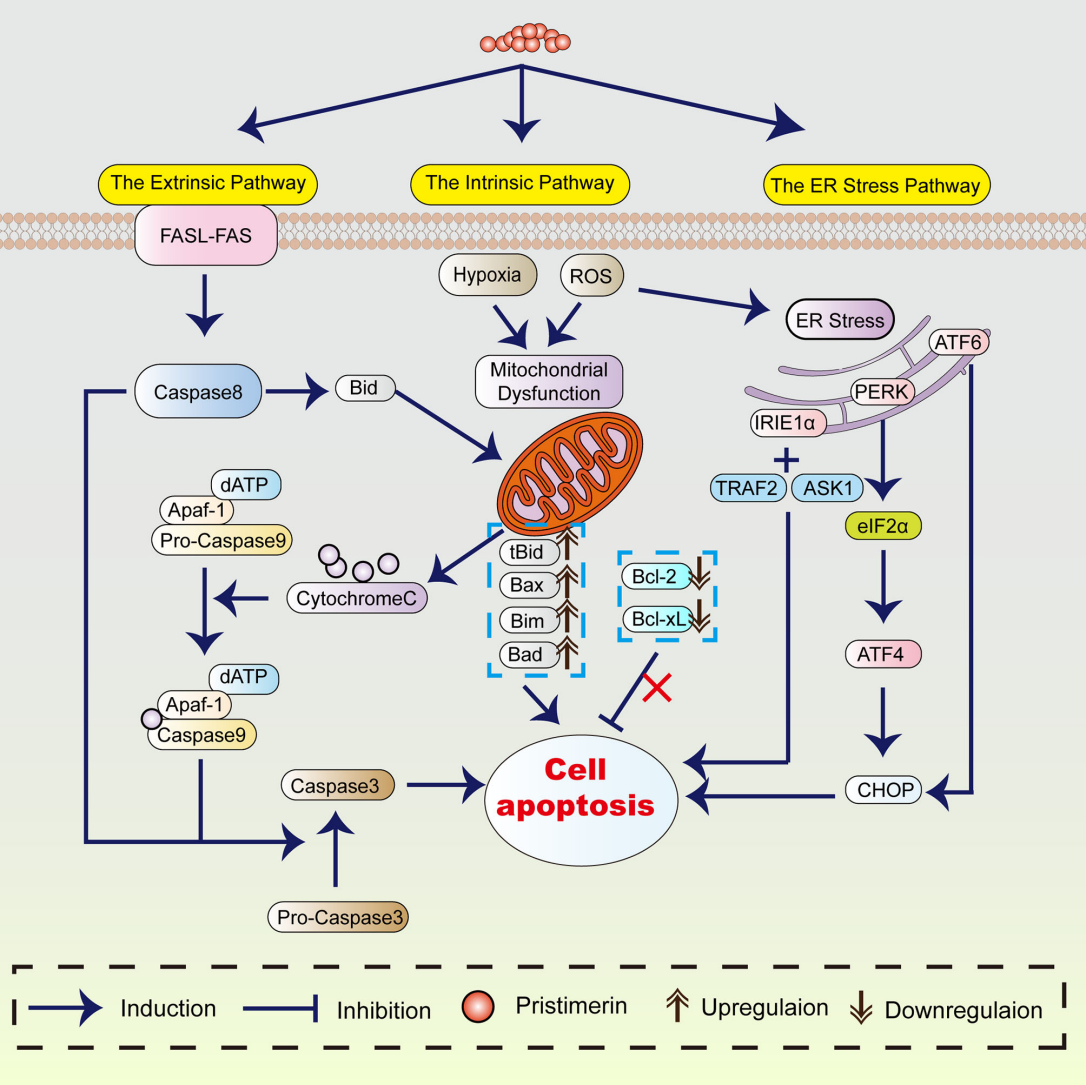
The mechanism of pristimerin on apoptosis. Pristimerin induces apoptosis through the intrinsic (mitochondrial), extrinsic (death receptor), and ER stress pathways.

Apoptosis refers to programmed and ordered cell death, maintaining the cell and body homeostasis. As early as the 1970s, a study showed that apoptosis was crucial to eliminating potentially malignant cells, hyperplasia, and tumor progression (25). Thus, apoptosis induction plays an essential role in cancer treatment. The proposed mechanism of pristimerin on tumor cell apoptosis has been summarized in **Figure 1**.

#### Morphological Alterations in Apoptosis

Considerable evidence has revealed that under pristimerin treatment, morphological changes associated with apoptosis occur in UM (18), breast cancer (21, 49, 50), CML (39), hepatocellular carcinoma (HCC) (51), and glioma (52). Pristimerin has been reported to induce chromatin condensation and nuclei fragmentation accompanied by cell shrinkage. These morphological alterations are recognized as the hallmarks of apoptosis, which can be triggered by the intrinsic (or mitochondrial), extrinsic (or death receptor), and endoplasmic reticulum pathways (53).

#### The Intrinsic (Mitochondrial) Pathway

The intrinsic mitochondrial pathway is initiated within the cell. There are various internal stimulating factors inducing apoptosis such as ROS (18, 39, 51, 52, 54–56), and hypoxia (31). Pristimerin could promote ROS generation and lead to the loss of mitochondrial membrane potential, suggesting the occurrence of mitochondrial dysfunction (9, 30, 38, 50, 55–57). In contrast to the ROS-dependent apoptosis, a study has stated that pristimerin exhibits activities directly on mitochondria instead of accumulating ROS, subsequently induces mitochondrial dysfunction in MDA-MB-231 human breast cancer cells (49).

In the mitochondrial pathway, cytochrome-c, Apaf-1, and other apoptotic factors are released into cytoplasm due to the elevated mitochondrial permeability (58, 59), which is preceded caspase activation and cell apoptosis (53). The translocation of cytochrome-c from mitochondria into cytoplasm has been observed under pristimerin administration in CRLC (19), CML (43), breast cancer (49), HCC (51), glioma (52), prostate cancer (55), cervical cancer (56), and pancreatic cancer (60). Wu et al. have shown that pristimerin can induce cytochrome-c release through a direct action on mitochondria (49). Cytochrome-c in the cytoplasm will combine with Apaf-1, and caspase-9 to form apoptosome and then activate caspase-3 to initiate the caspase cascade, resulting in the cleavage of PARP. Mounting studies have shown that pristimerin activates caspase-3, followed by the cleavage of PARP in UM (18, 30), breast cancer (21, 49), colitis-associated colorectal cancer (CAC) (22), OSCC (27), CRC (28), CML (43), glioma (52), prostate cancer (55, 61), and cervical cancer (56).

Overactivation of PARP-1 prompts the transference of apoptosis-inducing factor (AIF) from mitochondria into the nucleus, contributing to cell apoptosis (62). The release of cytochrome-c and AIF has been observed in imatinib-resistant CML cells after pristimerin medication (43). However, Zhao et al. have discovered that pristimerin-treated U251 human glioma cells manifest morphological features including electron-lucent cytoplasm, loss of plasma membrane integrity, and intact nuclear membrane consistent with programmed necrosis rather than apoptosis (54). They have concluded that AIF-dependent programmed necrosis rather than caspase-dependent apoptosis is the main mechanism in glioma cells treated by pristimerin (54).

BCL-2 protein family serves as the regulator of the intrinsic apoptotic pathway (63), including pro-apoptotic (e.g., Bax, Bak, Noxa) and anti-apoptotic (e.g., Bcl-2, Bcl-xL, Mcl-1) proteins (64, 65). Pristimerin has been reported to attenuate the levels of Bcl-2 and Bcl-xL whereas enhance Bax expression in pancreatic cancer (41), cholangiocarcinoma (42), fibrosarcoma (66), and colon cancer (38). However, Wu et al. have found that pristimerin does not dramatically impact the expression of BCL-2 family proteins during apoptosis in MDA-MB-231 human breast cancer cells (49). Noxa/Mcl-1 complex can be formed when Noxa is translocated into mitochondria and is closely related to the discharge of cytochrome-c and caspase reactions (65, 67). Pristimerin can stimulate the coaction between Noxa and Mcl-1, consequently contributing to enhanced apoptosis in CRC (28). The inhibitor of apoptosis proteins (IAPs) also plays a central role in apoptosis modulation, including NAIP (BIRC1), c-IAP1 (BIRC2), c-IAP2 (BIRC3), X-linked IAP (XIAP, BIRC4), Survivin (BIRC5), Apollon (BRUCE, BIRC6), Livin/MLIAP (BIRC7) and IAP-like protein 2 (BIRC8) (68). In CRLCs, pristimerin upregulated the levels of Bax, whereas downregulated the levels of Bcl-2 and BIRC6. As a result, cytochrome-c was released from mitochondria accompanied by the attenuation of MMP, eventually intensifying the pristimerin-induced mitochondrial apoptotic pathway (19). Zhang et al. have revealed that in treatment with pristimerin, the decline of XIAP and survivin rather than Bcl-2 and Bcl-xL can be observed in UM cells (30). Similar to LNCaP and PC-3 prostate cancer cells (25), the overexpressed survivin reduced the chemo-sensitivity of UM cells, which can be reversed by pristimerin (30). In CML cells harboring T315I mutation, pristimerin downregulated the expression of Bcl-xL and Mcl-1 without any significant changes in Bcl-2, XIAP, Bax and survivin (43). Pristimerin treatment weakened the expression of Bcl-2 and survivin but did not markedly affect the levels of Bax in MiaPaCa-2 and Panc-1 pancreatic ductal adenocarcinoma (PDA) cells (60). Besides, pristimerin could also inhibit the expression of Bcl-2, Bcl-xL, survivin, XIAP, cIAP-1 and induce the expression of Bax, Bak and Bad in prostate cancer (25). Considering these results, the alterations of apoptosis-regulatory factors are diverse in various pristimerin-treated cancers. What these findings have in common is that pristimerin enhances the expression of pro-apoptotic proteins whereas abrogates the expression of anti-apoptotic proteins to promote apoptosis.

#### The Extrinsic (Death Receptor) Pathway

The extrinsic pathway is induced by the binding of the death ligands (e.g., TNF-α, FasL, TRAIL) with their death receptors, activating caspase-8 and caspase-3, contributing to apoptosis. So, the convergence of the intrinsic and extrinsic apoptosis pathways is caspase-3. The cleavage of caspase-8 and caspase-3 has been found in K562 CML cells under pristimerin treatment (39). Besides, emerging studies have demonstrated that pristimerin activates both the intrinsic and extrinsic apoptotic pathways in breast cancer (32, 69), pancreatic cancer (60), ovarian cancer (57), osteosarcoma (70), colon cancer (38). The activation of caspase-9, caspase-8, caspase-3, and cleaved PARP can be observed.

#### The Endoplasmic Reticulum (ER) Pathway

The ER pathway is closely associated with pristimerin-induced apoptosis. ER stress initiates unfolded protein response (UPR) to decrease stress and regain ER homeostasis, which is considered an adaptive way in cancer (71). However, if ER stress becomes severe and sustained, UPR will switch from a pro-survival mode into a pro-death response, resulting in intrinsic mitochondrial apoptosis (72). Hitomi et al. have found that caspase-4 is primarily activated in ER stress-induced apoptosis and causes the activation of caspase-9 (73). Pristimerin has been reported to induce the cleavage and activation of caspase-4 in CRLCs (19). The knockdown of caspase-9 and caspase-3 did not affect caspase-4 activity, whereas the inhibition of caspase-4 abrogated the activation of caspase-9 and caspase-3 (19), suggesting the critical role of caspase-4 in pristimerin-induced caspase cascade.

ER stress causes the dissociation of inositol requiring kinase1α (IRE1α), protein kinase-like ER kinase (PERK), and activating transcription factor 6 (ATF6). The activation of the ER stress/IRE1α signaling can upregulate C/EBP homologous protein (CHOP), which induces apoptosis (74). A recent study has demonstrated that the inhibition of IRE1α attenuates the induction of cleaved PARP, caspase-3 and Noxa in HCT116 and SW620 CRC cells, indicating that IRE1α is involved in the caspase-dependent apoptosis (28). Additionally, IRE1α can recruit TNFR-associated factor 2 (TRAF2) and apoptosis signal-regulating kinase 1 (ASK1) to form the IRE1α-TRAF2-ASK1 complex, activating the JNK signaling and leading to apoptosis consequently (21, 75). It has been revealed that pristimerin not only promotes the activation of TRAF2 and ASK1 but also enhances the interaction of IRE1α with TRAF2 and ASK1 in CRC (28). Besides, the upregulation of various ER stress-associated proteins, such as CHOP, GRP78, ATF4, ATF3, p-eIF2α, and p-IRE1α, has also been observed in CRLCs (19) and U266 and H929 myeloma cells (76).

### Autophagy Induction

As a lysosomal self-digestion pathway, autophagy is closely associated with the maintenance of cell homeostasis and prevention of tumorigenesis *via* eliminating unfunctional proteins and organelles. Thus, inducing autophagy in cancer cells is an important strategy for treatment (77, 78).

Autophagy-related genes (ATGs) are involved in the formation of the autophagosome (79), encoding ATG proteins. In conjugation with ATG7, ATG4B conjugates LC3I and PE to form LC3II (also known as MAP1LC3B) and the LC3 conversion (LC3-I to LC3-II) is recognized as a hallmark of autophagy (80). Upon exposure to pristimerin, the accumulation of autophagosome was elevated and the ratio of LC3-II/LC3-I was increased in Eca109 esophageal cancer cells (40) and QBC and RBE cholangiocarcinoma cells (42). The interaction between Beclin-1 and other cofactors (e.g., Atg14L, PINK and survivin), which is essential in the modulation of the lipid kinase Vps-34 protein and formation of Beclin-1-Vps34-Vps15 core complexes, can result in the occurrence of autophagy (81). Pristimerin has been reported to elevate the expression of Beclin-1, ATG7, and LC3-II in K562 CML cells (39). Furthermore, the transcription of p62 is also significantly augmented during the process of autophagy (82). Zhao et al. have found that pristimerin increases the expression of p62 and the levels of LC3-II in MDA-MB-231, MDA-MB-468, and MCF-7 human breast cancer cells (21, 50).

The relationship between autophagy and apoptosis has been investigated in several studies. It has been demonstrated that the suppression of autophagy inhibits pristimerin-induced cell viability loss and apoptosis, indicating autophagy contributes to apoptosis. Moreover, the attenuation of caspase reduced the expression of LC-II, suggesting the inhibition of apoptosis suppressed autophagy (21, 39).

### The Inhibition of Cell Migration, Invasion, and Metastasis

The metastasis of malignancies is the final step of the invasion metastasis cascade, a subsequent cell-biological event of disseminating from the primary tumor and entering distant organs and adjusting to remote tissue microenvironments (83, 84).

#### Cell Adhesion and Cytoskeleton Inhibition

As modulators of the tumor microenvironment, the matrix metalloproteinases (MMPs) play a significant role in carcinoma progression (85). Mounting evidence has shown that pristimerin can downregulate the levels of MMP2 and MMP9, indicating the inhibition of cell migration in H1299 lung cancer cells (29), esophageal squamous cell carcinoma (ESCC) cells (86), UM cells (30), and CRLCs (19). Additionally, pristimerin has been found to decrease the expression of vimentin, F-actin, integrinβ1, and other cytoskeleton polymers, which are essential in cell migration and adhesion process in H1299 lung cancer cells (29).

#### Epithelial-Mesenchymal Transition (EMT) Inhibition

Epithelial-mesenchymal transition (EMT) refers to the transformation from stationary epithelial cells to mesenchymal cells, providing tumor cells with migration and invasion characteristics (87, 88). EMT is known as the hallmark in cancer metastasis (87). It has been revealed that pristimerin decreases the expression of EMT-related proteins, including N-cadherin, fibronectin, vimentin, and ZEB1 in PC-3 prostate cancer cells (31). Furthermore, the knockdown of EMT upstream protein Snail has been observed in H1299 lung cancer cells under pristimerin administration (29).

#### Cancer Stem Cells (CSCs) Inhibition

Cancer stem cells contribute to tumorigenesis, tumor metastasis and chemo-resistant (89). The CSCs characteristics contain the activation of aldehyde dehydrogenase (ALDH), the formation of tumorsphere, and the expression of surface markers including CD44, CD133 (89, 90). It has been suggested that pristimerin suppresses the self-renewal capability and characteristics of CSCs in ESCC (86), UM (30), and prostate cancer (31, 91), confirmed by the reduction of the percentage of ALDH+ cells and the inhibition of tumorsphere formation. In UM, stemness-related proteins, including Slug and Sox2, were downregulated, while the expression of Nanog and KLF4 was constant under pristimerin treatment (30). The expression of CD44, CD133 and other stemness factors such as KLF4, OCT4, and AGO2 were attenuated under pristimerin treatment in PC-3 prostate cancer (31, 91). Furthermore, the elimination of CSCs by pristimerin might be associated with NF-κB pathway in ESCC cells (86).

#### Angiogenesis Inhibition

Angiogenesis refers to a complex process orchestrating the formation of new capillaries from previous vessels (92), which is central to tumor migration, invasion, and metastasis (93). The interaction between vascular endothelial growth factor (VEGF) and VEGFR can facilitate pathological angiogenesis (94). It has been revealed that pristimerin blocks VEGF-triggered angiogenesis in human umbilical vascular endothelial cells (HUVECs) (95) and PC-3 prostate cancer cells (91). The proliferation and tube formation of endothelial cells are two crucial steps of angiogenesis (95). Pristimerin has been found to inhibit VEGF-induced angiogenesis by suppressing cell proliferation, chemotactic motility, and tube formation (19, 95).

## Molecular Mechanisms Involved in Anti-Cancer Activities of Pristimerin

Molecular signal systems are complex, containing various signal factors and pathways that are cross-talk with each other. The interaction of various signaling components plays a critical role in tumorigenesis. Thus, pristimerin exhibits anti-cancer actions *via* regulating a wide variety of signal targets and pathways.

### Effects on EGFR/HER2: Modulating Downstream PI3K/AKT, MAPK Pathways and Inhibiting Cell Invasion, Metastasis, Angiogenesis

Epidermal growth factor receptor (EGFR) family consists of four related proteins including EGFR, HER2, HER3 and HER4 (96). The binding of specific ligands to these receptors stimulates the formation of homodimer (EGFR-EGFR) or heterodimer (EGFR-HER2, EGFR-HER3, or EGFR-HER4), and subsequently activates downstream PI3K/AKT and MAPK pathways (97). Mounting evidence has confirmed the overexpression of HER2 and the significance of HER2-targeted therapy in various cancers such as breast cancer, colon cancer, etc (98, 99). Pristimerin has been reported to abrogate the expression of EGFR and its downstream factors such as p-Akt, p-ERK1/2 in HepG2 HCC cells (51) and U87 glioma cells (52). Yousef et al. have revealed that pristimerin inhibits the phosphorylation of EGFR and HER2 in colon cancer cells, followed by the suppression of downstream ERK1/2, Akt, mTOR and NF-κB signaling pathways (38). In HER2 -positive SKBR3 human breast cancer cells, overexpression of HER2 could stimulate the expression of fatty acid synthase (FASN) through PI3K/AKT or MAPK pathways, leading to tumorigenesis. Pristimerin has been found to repress the expression of FASN *via* the inhibition of HER2 (69). Furthermore, it has also been demonstrated that HER2 overabundance enhances cell invasion, metastasis, and angiogenesis by stimulating mTOR/p70S6K pathway and elevating MMP2 and MMP9, which can be diminished by pristimerin administration (69).

### Effects on IGF-1R: Regulating Downstream PI3K/AKT, ERK/MAPK Pathways and Resulting in G1 Phase Arrest

Insulin-like growth factor (IGF) signaling system is closely related to cell homeostasis maintenance. During tumorigenesis, the IGF-1 receptor is commonly upregulated in cancer cells and affects the proliferation, migration, and metastasis of the malignancies (100). The IGF-1R system is also associated with other downstream signaling ways such as PI3K/AKT and ERK/MAPK pathways (101, 102). Xie et al. have suggested that pristimerin inhibits IGF-1R/Akt/mTOR and ERK1/2 pathways and consequently leads to the loss of UM cell viability. They have also found that IGF-1 stimulates the expression of cyclin D1, which can be reversed by pristimerin to induce G1 phase arrest (23).

### Effects on PI3K/AKT Pathway: Inducing Apoptosis and Inhibiting Cell Invasion, Migration, Angiogenesis, Metastasis

The PI3K/AKT/mTOR (phosphatidylinositol 3-kinase/Protein Kinase-B/mechanistic target of rapamycin) transduction pathway is of significance in modulating cell growth, motility, survival, metabolism, and angiogenesis (103). Phosphorylation of Akt can induce the downstream phosphorylation of NF−κB, mTOR, and FoxO3a, mediating cell growth, proliferation, survival, and angiogenesis. Numerous studies have shown that pristimerin inhibits PI3K/AKT pathway evidenced by the dephosphorylation of Akt and its downstream factors such as mTOR, FoxO3a, and NF−κB in UM (18), OSCC (27), breast cancer (32, 50), pancreatic cancer (60), ovarian cancer (57), osteosarcoma (70), and fibrosarcoma (66). The downregulation of mTOR-regulated p-S6K1 and p-4E-BP1 levels have been observed in MiaPaCa-2 and Panc-1 PDA cells (60) and HCT-116 CRC cells (104). Furthermore, pristimerin has been reported to decrease the phosphorylation of Akt and FoxO3a, inducing nuclear accumulation of FoxO3a in UM (18) and CAC (105).

It is well known that PI3K/AKT pathway is associated with various cellular processes. Yan et al. have observed that the attenuation of Akt enhances the upregulation of the pro-apoptotic molecules (BIM, p27Kip1, cleaved caspase-3, PARP and Bax) and suppression of anti-apoptotic proteins (cyclin D1 and Bcl-2), consequently potentials pristimerin-induced apoptosis in UM-1 cells (18). Besides, pristimerin has also been proved to downregulate the expression of FoxO3a targeted genes cyclinD1 and Bcl-xL while augmenting the expression of p21 and p27 (105), confirming the stimulation of apoptosis in AOM/DSS treated colon tissue. In CRC, the inhibition of P70S6K/4EBP1 has been observed to repress cancer cell invasion, migration, angiogenesis, and metastasis (104). According to these findings, it can be concluded that pristimerin induces cell apoptosis and inhibits cell migration, invasion, and metastasis by impairing PI3K/AKT pathway.

### Effects on NF-κB Pathway: Inducing Apoptosis and Inhibiting Inflammation, Proliferation, Angiogenesis, Invasion

NF-κB transcription factors are increasingly considered the critical regulators of cancer initiation and progression (106). Various signaling pathways such as Ras/MAPK and PI3K/Akt are involved in the stimulation of NF-κB pathway, resulting in phosphorylation, ubiquitination, and degradation of IκBα, p65 translocation and overexpression of NF-κB-dependent gene including cyclin D1, Bcl-2 and Bcl-xL (107).

Pristimerin inhibited the expression and translocation of p65, the phosphorylation of IκBα and the activation of IKKα/β, identifying the inhibition of constitutive NF-κB pathway in CRC (20), pancreatic cancer (41), CML (43), ESCC (86), and multiple myeloma tumors (108). Besides, TNF-α/LPS-induced NF-κB pathway was also prevented upon pristimerin treatment. Numerous studies have shown that pristimerin inhibits the levels of p-IKK, p-IκBα, and p65 translocation induced by TNF-α or LPS (20, 30, 86). These findings have come to a consensus that pristimerin inhibits both classical and induced NF-κB pathways in various cancers. Moreover, Lu et al. have found that pristimerin represses the DNA binding of NF-κB in intact cells instead of directly inhibiting in a purified nuclear extract (43).

NF-κB-dependent gene expression was also abrogated by pristimerin. Accumulating evidence has demonstrated that pristimerin markedly decreases NF-κB-dependent gene expression including antiapoptotic (Bcl-2, Bcl-xL, survivin, Mcl-1, c-IAPl), invasive (MMP9), cell survival (cyclin D1, c-Myc), angiogenic (VEGF), and proinflammatory (COX-2, iNOS) genes (20, 30, 41, 43, 57, 60, 86), thus affects corresponding cellular events in various cancers. The pro-inflammatory cytokines IL-6 and TNF-α were also downregulated due to the suppression of NF-kB pathway in CAC (105). Furthermore, it has been reported that the inhibition of the Akt/mTOR pathway and NF-κB can enhance the effects of anti-cancer drugs and radiation and suppresses tumor angiogenesis in osteosarcoma cells (70). Likewise, the inhibition of NF-κB may account for the promoted chemosensitivity to gemcitabine in pancreatic cancer cells (41).

### Effects on ROS Generation and MAPK Pathway: Inducing Apoptosis and Autophagy

Accumulating evidence has shown that excessive ROS under pristimerin treatment leads to mitochondrial permeability augmentation and mitochondrial membrane potential reduction in colorectal cancer (104), prostate cancer (55), glioma (52), HCC (51), and UM (18). The results of studies have indicated that ROS may induce cell apoptosis *via* ROS-mediated mitochondrial dysfunction. Uncontrolled ROS generation also triggered ER stress and caused the accumulation of unfolding protein, resulting in ER stress-dependent apoptosis in CRC (28). Furthermore, G0/G1 phase arrest induced by pristimerin might be associated with ROS accumulation in UM (18) and breast cancer (21).

Mitogen-activated protein kinases (MAPKs) include three major subfamilies: the extracellular signal-regulated kinases (ERK); the c-Jun NH2-terminal kinase or stress-activated protein kinases (JNK); and p38 (109). Pristimerin has been reported to increase the levels of p-JNK and p-p38, whereas decrease the levels of p-ERK in MDA-MB-231 and MDA-MB-468 breast cancer cells (21), and K562 CML cells (39). It has been revealed that cell viability loss can be reversed by JNK inhibitor but not p38 inhibitor, indicating that JNK activation results in cell death in breast cancer, CML and cervical cancer (21, 39, 56). ROS is closely associated with JNK activation and is generated by pristimerin in various ways. Zhao et al. have found that the inhibition of Trx-1 caused by pristimerin leads to ROS accumulation, and then induces the phosphorylation of ASK1 and JNK, resulting in cell death in breast cancer (21). Another study has demonstrated that pristimerin improves intracellular ROS levels through both stimulating excessive generation of superoxide in mitochondria and abrogating intracellular ROS scavenger GSH level in glioma cells, consequently activates JNK (54).

Numerous studies have shown that the activation of ROS/JNK pathway induces pristimerin-induced apoptosis by elevating the expression of Bax, Bim, cleaved PARP and caspase-3; reducing the expression of Bcl-2, Bcl-xL, XIAP and MMP; decreasing mitochondrial transmembrane potential and promoting the nuclear accumulation of AIF (54, 56, 66, 86, 104). The relationship between ROS/JNK pathway and apoptosis can also be proved by NAC (an ROS scavenger) and JNK inhibitor. In K562 CML cells, NAC or JNK inhibitor abrogated pristimerin-induced expression of apoptosis-related protein and LC3B II, confirming the critical role of ROS/JNK in apoptosis and autophagy (39). Furthermore, another study has shown that JNK inhibitor inhibits cleaved PARP, caspase-3 and noxa, thus attenuates pristimerin-induced apoptosis in HCT116 and SW620 CRC cells (28). Based on these studies, it can be concluded that pristimerin increases the levels of intracellular ROS and triggers ROS/JNK pathway to strengthen apoptosis and cell death.

### Effects on EphB4/CDC42/N-WASP: Inducing Apoptosis and Inhibiting Angiogenesis

The interaction between erythropoietin-producing hepatoma (Eph) receptors and their Eph receptor-interacting (ephrin) ligands has a central role in cell communication. EphB4/ephrinB2 is associated with tumor angiogenesis, growth, and metastasis (110, 111). Tang et al. have concluded that the impaired activation of EphB4 by pristimerin can result in caspase-4/-9 activation, ER stress-associated protein (CHOP, GRP78, ATF4, p-eIF2α, and p-IRE1α) upregulation, antiapoptotic Bcl-2 members downregulation, ROS production, and MMP loss, suggesting the induction of mitochondrial and ER-stress apoptotic pathways in CRLCs (19). Besides, the knockdown of EphB4 repressed the activation of ephrinB2, thereby inhibited cell migration and capillary formation (19), confirming the critical role of ephrinB2 in angiogenesis.

CDC42 and N-WASP command a vital position in cell adhesion, cytoskeleton formation and cell cycle regulation (112). It has been revealed that pristimerin downregulates the levels of CDC42 and N-WASP in CRLCs, accompanied by inducing ROS generation, ER stress and increasing Bax/Bcl-2 ratio (19). Therefore, pristimerin induced apoptosis and inhibited angiogenesis in CRLCs *via* the suppression of EphB4/CDC42/N-WASP pathway.

### Effects on Wnt/β-catenin Pathway: Inducing G1 Phase Arrest, Autophagy, and Inhibiting Inflammation

The Wnt/β-catenin signaling pathway plays a significant role in cell development, growth, and differentiation. The aberrant activation of this pathway results in many malignancies. Pristimerin may induce G1 phase arrest and inhibit inflammation in CAC, given that Wnt target genes including c-Myc, cyclinD1, and cox-2 were downregulated in HCT116 and HT-29 CRC cells (22). Another study has also found that pristimerin inhibits Wnt signaling and triggers incomplete autophagy in MCF-7s breast cancer cells proved by the reduction of total β-catenin levels and LRP6 expression/phosphorylation, and the elevation of LC3-II levels (50).

Dishevelled (Dvl), a positive regulator in Wnt/β-catenin signaling, increases β-catenin levels through the GSK3β phosphorylation/inactivation and promotes the binding of activated Akt with the Axin-GSK3β complex (113). Cevatemre et al. have shown that the expression of Dvl3 is decreased in pristimerin-treated MCF-7s breast cancer cells (50). In CRC, the degradation of β-catenin by GSK3β activation may represent the critical mechanism underlying the pristimerin-induced cell death (22). The levels of p-Akt were also decreased in MCF-7s breast cancer cells, confirming the inhibition of PI3K/AKT pathway was associated with the inhibition of Wnt/β-catenin pathway in breast cancer under pristimerin treatment (50).

### Effects on Shh/Gli1 Pathway: Inhibiting Angiogenesis

Abnormal activation of sonic hedgehog (Shh)/Glioma-associated oncogene homolog I (Gli1) pathway occurs in human neoplasms. The Shh signaling pathway is divided into canonical and non-canonical pathways. In the canonical pathway, Shh ligands, produced by tumors, eliminate the suppression of Smoothened (SMO) by Patched (PTCH), resulting in activation of Gil, which can modulate downstream gene expression and affect cell survival, growth, invasion, and migration. The non-canonical pathway is recognized as the alternative pathway to the canonical one (114).

A recent study has shown that pristimerin exerts inhibitory effects on tumor angiogenesis through suppressing Shh/Gli1 and its downstream pathways in non-small cell lung cancer (NSCLC). Pristimerin treatment has been reported to inhibit Shh-induced endothelial cellular essential processes during angiogenesis, with a concomitant abrogation of Shh-induced pericytes recruitment to the new blood vessels which is crucial for the maturation of blood vessels. Besides, Gli1 nucleus distribution in endothelial cells and pericytes was also repressed upon pristimerin treatment (17).

As mentioned above, the activation of VEGFR2 promotes angiogenesis. It has been revealed that Shh activation elevates the levels of phosphorylated VEGFR2 and pristimerin attenuates Shh-induced the phosphorylation of VEGFR2 (17), implying the inhibition of downstream VEGF/VEGR2 pathway is correlated with the suppression of Shh/Gil1 pathway to exert anti-angiogenic activities.

### Effects on HIF-1α/SPHK-1 Pathway: Inducing G1 Phase Arrest and Inhibiting Angiogenesis, CSCs, EMT

Hypoxia is a common property of locally advanced solid tumors, resulting in metastatic progression and chemoresistance with the involvement of hypoxia-inducible transcription factors (115, 116). The transcription factor hypoxia-inducible factor 1α (HIF-1α) is overexpressed in human cancers due to intra-tumoral hypoxic conditions, leading to angiogenesis, survival, and proliferation of tumor cells (117). Pristimerin has been reported to inhibit the expression of HIF-1α and hypoxia-induced progression in hypoxic PC-3 prostate cancer cells (24, 31). Lee et al. have revealed that in the presence of pristimerin, the decrease of cell viability under hypoxia is markedly lower than that under normoxia, implying that HIF-1α contributes to tumor resistance (24). Besides, pristimerin also inhibited hypoxia-induced sphere formation, colony formation, expression of CSC markers and stemness factors (CD44, KLF4, OCT4, AGO2), as well as the expression of EMT markers (N-cadherin, fibronectin, vimentin, ZEB1) in PC-3 cells (31).

HIF-1α is modulated by sphingosine kinase 1(SPHK1) and its stabilization depends on AKT/GSK-3β which is another downstream of SPHK-1. The activity of SPHK-1 can be enhanced under hypoxia conditions and regulated by ROS (118). Pristimerin has been reported to downregulate the expression of HIF-1α *via* inhibiting SPHK-1 pathway. Meanwhile, the inhibition of AKT/GSK-3β was also associated with the attenuation of HIF-1α in prostate cancer (24).

Furthermore, the inhibition of SPHK-1 prevented the generation of VEGF and the expression of cyclinD1 and CDK4, indicating that pristimerin administration inhibited angiogenesis and induced G1 phase arrest in prostate cancer cells (24).

### Effects on Micro RNA: Exerting RNA Silencing Actions

Micro RNA (miRNA) is a large family of small non-coding RNA with about 22 nucleotides targeting the RNA-induced silencing complex (RISC), which acts as RNA silencers and post-transcriptional regulators of gene expression (119–121). AGO2 is closely related to miRNA-mediated gene silencing (122). The elevation of AGO2 levels was observed in glioma and breast cancer cells, suggesting the involvement of RNA silencing effects of miRNA upon pristimerin treatment (9, 123). Surprisingly, the expression of miR-542-5p, a mature sequence formed from pre-miR-542, has manifested different changes in glioma and breast cancers. Cheng et al. have found that miR-542-5p expression is increased after exposure to pristimerin, concomitant with the reduction of breast cancer cell viability (9). However, in glioma cells, the levels of miR-542-5p were decreased (123). Cheng et al. have considered that miR-542-5p may inhibit breast cancer cell proliferation (9), whereas Li et al. have demonstrated the cell survival promotion effects of miR-542-5p in glioma (123), according to their experiment results, respectively. Despite the differences in miR-542-5p expression, they both suggested that miR-542-5p might directly modulate AGO2 into RISC, and subsequently exert RNA silencing effects in tumors to inhibit the expression of DUB3 and PTPN1.

DUB3 exerts oncogenic potential by stabilizing the Cdc25A protein, which is a critical regulator of cell cycle progression, and its knockdown results in reduced Cdk/Cyclin activity and cell cycle arrest (124, 125). In breast cancer, miR-542-5p stimulated the formation of RISC to decrease the levels of DUB3. Pristimerin exhibited G1 phase arrest and tumor progression inhibition *via* miR-542-5p/DUB3 axis (9). In addition to DUB3, the protein tyrosine phosphatase nonreceptor type 1 (PTPN1) was also involved in the anti-tumor effects of miR-542-5p. PTPN1 has been reported to strengthen the progression of glioma by activating the MAPK/ERK and PI3K/AKT pathways (126). Cheng et al. have demonstrated that miR-542-5p can contribute to the cleavage of PTPN1 mediated by modulation of AGO2 and RISC (123).

### Effects on Proteasome: Inducing Apoptosis

As the most critical intracellular protein degradation system, the ubiquitin-proteasome pathway commands a vital position in maintaining cellular protein levels and coordinating basic cellular activities, such as cell growth, cell apoptosis, cell cycle control, DNA repair, and antigen presentation (127, 128).

The 26S eukaryotic proteasome is a catalytic complex composed of a 20S catalytic particle and two 19S regulatory particles. Three catalytic sites are presenting in the 20S core particle, including sites with chymotrypsin-like (β5), trypsin-like (β2), and peptidyl-glutamyl peptide-hydrolyzing (PGPH)-like or caspase-like (β1) activities (61, 129).

It has been reported that the interaction between conjugated ketone carbon(C6) of pristimerin and the N-terminal threonine of the proteasomal β5 subunit inhibits chymotrypsin-like activity and causes the accumulation of polyubiquitinated proteins and three proteasome target proteins (Bax, p27 and IκBα), consequently blocked the NF-kB pathway and facilitated cell apoptosis (61, 76, 130).

As an apoptotic inhibitor, survivin is overexpressed in malignancies, promoting cancer cell survival as well as inhibiting cell death (131). Pristimerin has been reported to downregulate survivin levels *via* the ubiquitin-proteasome degradation pathway, leading to tumor cell apoptosis in prostate cancer (25). Likewise, pristimerin inhibited the anti-apoptotic Bcl-2 and induced apoptosis in prostate cancer cells through ROS-dependent ubiquitin-proteasomal degradation pathway (55). Another study has also revealed that pristimerin suppresses the invasive abilities of breast cancer cells by suppressing proteasomal activity and increasing the levels of G protein signaling 4 (RGS4) (132).

### Effects on Telomerase: Abrogating Telomere Homeostasis

Mammalian telomeres consist of long-stranded hexamer TTAGGG nucleotide repeats and a related protein complex called shelterin (133, 134). The shelterin complex prevents end-to-end fusion and degeneration of chromosomes by forming the t loop (135). The TTAGGG repeat sequence shortens as each cell divides (136, 137). When telomeres become severely shortened, cellular senescence will be triggered, which is considered a tumor inhibition mechanism. However, telomerase is a reverse transcriptase that adds new DNA to the telomere at the end of the chromosome to maintain telomere homeostasis and inhibit growth arrest, which contains a catalytic protein subunit called telomerase reverse transcriptase (TERT), encoded by the hTERT gene. Thus, the upregulation of telomerase is a critical marker in cancers (138, 139).

Pristimerin has been found to inhibit the expression and activity of hTERT by downregulating the transcription factors such as Sp1, c-Myc, NF-κB, STAT-3 in pancreatic ductal adenocarcinoma (26) and prostate cancer (140). Due to the inhibition of telomerase activity, the telomere homeostasis can be broken and then cellular senescence occurs caused by shortened telomere. Pristimerin also impaired p-Akt, which could phosphorylate hTERT, indicating that the downregulation of phosphorylated hTERT by Akt contributed to the suppression of hTERT telomerase activity (26, 140).

## Pathways and Targets Involved in Chemoresistance Reversal

The characteristic that malignancies are cross-resistant to various drugs with different cell targets and structures is known as multiple drug resistance (MDR) (141). Nowadays, the largest challenge for cancer drug treatment is MDR initiated by the elevation of the ATP-binding cassette (ABC) efflux transporters, including P-glycoprotein (P-gp/ABCB1), multidrug resistance-associated protein 1 (MRP1/ABCC1) and breast cancer resistance protein (BCRP/ABCG2) (142, 143). Pristimerin has been reported to knockdown the expression of P-gp and reverse the chemoresistance mediated by ABCB1 in drug-resistant KBv200 cells (144). Moreover, it has been revealed that several targets and pathways are associated with drug resistance, such as PI3K/Akt signaling in Adriamycin-resistant breast cancer cells (32) as well as Bcr-Abl and NF-κB pathway in imatinib-resistant CML cells (43). Pristimerin could attenuate PI3K/Akt pathway, NF-κB pathway and Bcr-Abl to overcome the resistance (32, 43).

## Effective Synergy With Other Chemotherapeutic Drugs

**Figure 2:**
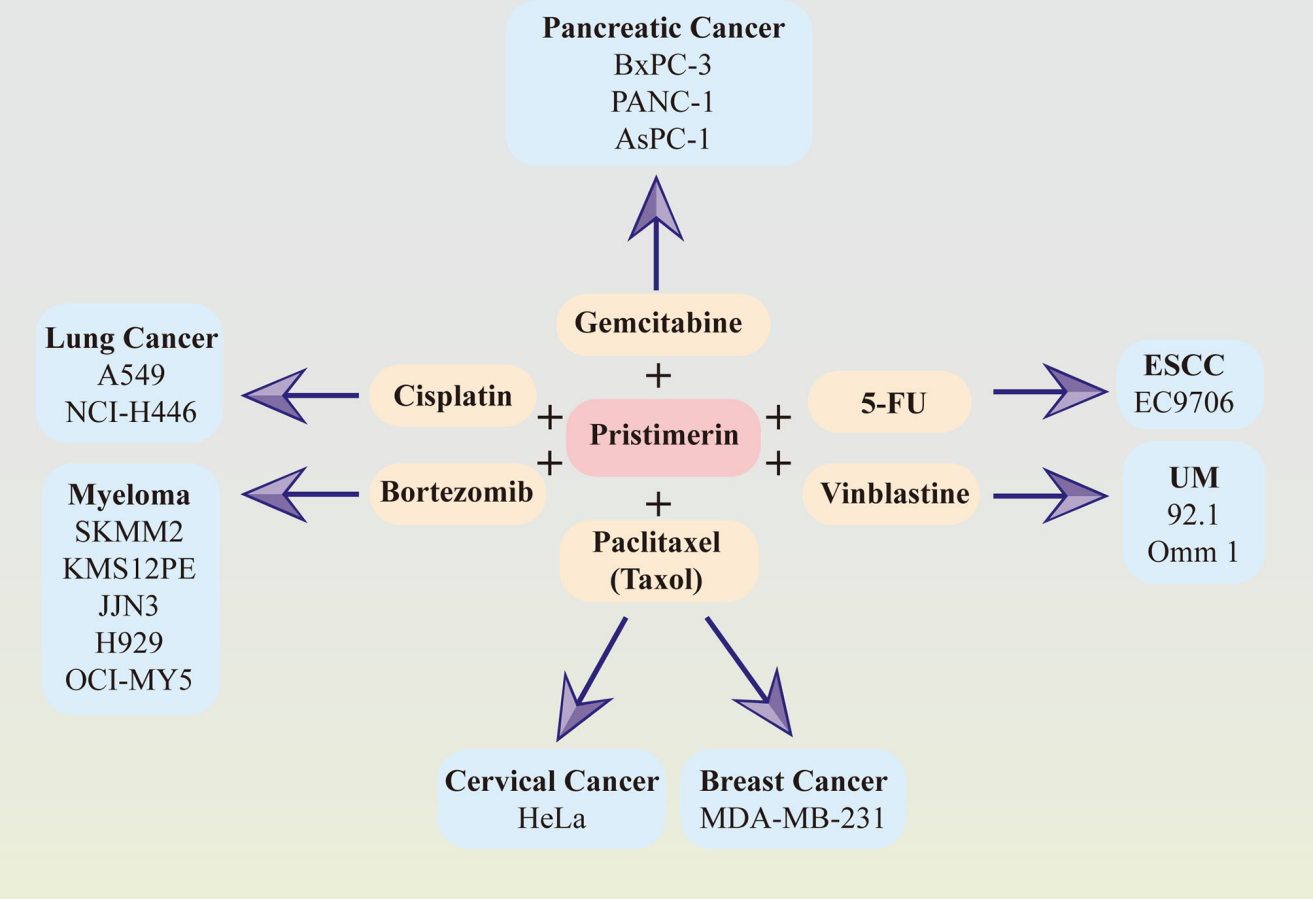
Synergy effects between pristimerin and other chemotherapeutic drugs. Pristimerin exhibits synergizing effects with other chemo-drugs, including gemcitabine in pancreatic cancer, paclitaxel (Taxol) in breast cancer and cervical cancer, cisplatin in lung cancer, bortezomib in myeloma, vinblastine in UM, and 5-FU in ESCC.

Considerable evidence has suggested that the combination of pristimerin with other chemo-drugs such as gemcitabine in pancreatic cancer (33, 41), paclitaxel(taxol) in breast cancer (34) and cervical cancer (130), cisplatin in lung cancer (145), bortezomib in myeloma (76), vinblastine in UM (30), 5-FU in ESCC (86). The synergy effects between pristimerin and other chemo-drugs have been illustrated in **Figure 2**.

Pristimerin may act as a chemosensitizer to enhance the therapeutic effects of gemcitabine *via* NF-κB signaling inhibition in pancreatic cancer cells (41). Another recent study related to pancreatic cancer has demonstrated that pristimerin synergizes with gemcitabine through attenuating Chk1/53BP1-mediated DNA repair (33). Paclitaxel, also known as taxol, synergized with pristimerin to enhance anti-tumor effects *via* ROS-mediated mitochondrial dysfunction in cervical cancer cells (130). The combination of them also induced autophagy *via* ERK1/2 inhibition in human breast cancer cells (34). In lung cancer cells, the effect of cisplatin was strengthened with pristimerin through the attenuation of the miR-23a/Akt/GSK3β signaling pathway and autophagy (145). It has also been observed that pristimerin can combine with vinblastine and exhibit a better therapeutic effect in UM (30) and synergize with 5-FU in ESCCs (86). Furthermore, the collaborative cytotoxicity between pristimerin and bortezomib has been reported in myeloma (76).

## The Effects of Pristimerin on Different Tumor Cell Lines and Models

Accumulating evidence *in vivo* and vitro has shown that pristimerin exhibits anti-cancer activities in different cancer cell lines and models. The anti-cancer activities and mechanisms of pristimerin have been summarized in **Table 1**.

**Table 1:** Anti-cancer activities of pristimerin in various cancer cell lines and models.

Cancer Types	Cell Lines	Cell Models	Mechanisms of action	References
Prostate cancer	PC-3	Intra-tibial injection mouse model	Inhibited bone metastasis by targeting PC-3 stem cell characteristics and VEGF-induced angiogenesis. Inhibited the bone destruction by the invasion of the tumor and reduced the tumorigenic potential of bone metastasis.	(91)
		——	Inhibited the hypoxia-induced proliferation, invasion, spheroid formation, colony formation, stem cell characteristics and EMT protein expression.	(31)
			Inhibited HIF-1α through the SPHK-1 pathway.	(24)
	LNCaP and PC-3	——	Induced apoptosis through ubiquitin-proteasomal degradation of antiapoptotic survivin.	(25)
			Inhibited hTERT mRNA expression, native and phosphorylated hTERT protein and hTERT telomerase activity attributed to the inhibition of transcription factors SP1, c-Myc and STAT3 and protein kinase B/Akt.	(140)
			Induced apoptosis in prostate cancer cells by down-regulating Bcl-2 through ROS-dependent ubiquitin-proteasomal degradation pathway.	(55)
	PC-3, LNCaP, and C4-2B	——	Induced apoptosis by targeting the proteasome and inhibited the proteasomal chymotrypsin-like activity.	(61)
Breast cancer	MDA-MB-231	——	Resulted in a rapid release of cytochrome c from mitochondria, which preceded caspase activation and the decrease of mitochondrial membrane potential but not significantly altered the protein level of Bcl-2 family members, nor did it induce Bax translocation.	(49)
		Human breast cancer xenograft model	Inhibited tumor migration and invasion by inhibiting proteasomal activity and increasing the levels of RGS4. Inhibited tumor growth and invasion.	(132)
		——	Combination of pristimerin and paclitaxel additively induced autophagy in human breast cancer cells *via* ERK1/2 regulation	(34)
	MDA-MB-231 and MDA-MB-468	MDA-MB-231 tumor xenografts in nude mice	Induced apoptosis and autophagy *via* activation of ROS/ASK1/JNK pathway. Significantly suppressed tumor volume and weight in the mice.	(21)
	MCF-7, MDA-MB-231, and 4T1	——	Inhibited breast cancer cell viability, migration, and cell cycle and induced cell apoptosis through upregulating miR-542-5p while downregulating DUB3.	(9)
	MCF-7	——	Overcomed Adriamycin resistance in breast cancer cells through suppressing Akt signaling.	(32)
	SKBR3	——	Decreased HER2 expression, fatty acid synthase and inhibited the Akt, MAPK, and mTOR signaling pathways to affect metastasis and apoptosis.	(69)
	HUVECs	Human breast cancer xenograft model	Reduced tumor volume and weight, inhibited tumor growth and angiogenesis associated with downregulation of VEGF.	(95)
	MCF-7 and MDA-MB-231	Human breast cancer xenograft model	Induced apoptosis and an incomplete autophagy Reduced tumor size and weight, insignificantly increased toxicity, and behavioral changes (e.g., dizziness) in an E/T80/WFI carrier compared to D/PBS.	(50)
Glioma	U373	——	Inhibited Glioma progression by targeting AGO2 and PTPN1 expression *via* miR-542-5p.	(123)
	U87	——	Induced cell apoptosis through ROS-mediated mitochondrial dysfunction.	(52)
		Human glioma xenograft model	Triggered AIF-dependent programmed necrosis in glioma cells *via* activation of JNK.	(54)
Colorectal cancer	HCT-116	AOM/DSS model of colitis-associated colorectal carcinogenesis	Inhibited the growth of cancer cells *via* inhibiting and targeting of AKT, and probably the downstream FOXO3a pathway. Decreased tumor burden.	(105)
		Human colorectal cancer xenograft model	Downregulated PI3K/AKT/mTOR pathway and its subsequent downstream p70S6K and E4-BP1 proteins. Inhibited tumor growth and induced apoptosis.	(104)
		Human colorectal cancer xenograft model	Inhibited NF-кB signaling pathway.	(20)
		Tumor xenograft in nude mice	Induced apoptosis through activation of ROS/ER stress-mediated noxa and elevated the expression of ER stress-related proteins, resulting in activation of the IRE1α and JNK signal pathway through the formation of the IRE1α-TRAF2-ASK1 complex. Significantly suppressed tumor volume.	(28)
	HCT-116, COLO-205 and SW-620	——	Downregulated the phosphorylated forms of EGFR and HER2 proteins, and subsequently caused a decrease in the phosphorylated forms of Erk1/2, Akt, mTOR and NF-κB.	(69)
	HCT116 and HT-29	Human colon carcinoma xenograft	Inhibited inflammatory responses and Wnt/β-catenin signaling Significantly inhibited the tumor volume.	(22)
Esophageal cancer	Eca109 and Ec9706	Tumor Xenograft model	Decreased the protein expression of CDK2, CDK4, cyclin E, and Bcl-2 and increased the expression of CDKN1B Elevated the ratio of LC3-II/LC3-I. Decreased tumor size and weight.	(40)
	EC9706, EC109, and KYSE30	Human ESCC xenograft model	Inhibited proliferation, migration, and invasion *via* suppressing NF‐κB pathway.	(86)
Leukemia	HL-60	——	Inhibited DNA synthesis.	(146)
	K562(CML)	——	Induced autophagy-mediated cell death through the ROS/JNK signaling pathway.	(39)
	KBM5 and KBM5-T315I	Imatinib-resistant Bcr-Abl-T315I xenografts in nude mice	Blocked the TNFα-induced IκBα phosphorylation, translocation of p65, and expression of NF-κB-regulated genes. Inhibited the expression of Bcr-Abl.	(43)
Uveal melanoma	RGC-5 and the D407	——	Induced apoptotic cell death through inhibition of PI3K/Akt/FoxO3a pathway.	(18)
	Human uveal melanoma cell lines	——	Inhibited the cell proliferation through downregulating phosphorylated IGF‐1R and its downstream signaling.	(23)
	92.1, Mel 270, Omm 1 and Omm 2.3	——	Inhibited the malignant phenotypes by inactivating NF−κB pathway.	(30)
Lung cancer	NCI-H1299(NSCLC)	——	Inhibit angiogenesis targeting Shh/Gli1 signaling pathway	(17)
			Decreased migration and invasion of H1299, which were correlated with EMT-related proteins and mRNA.	(29)
	Lung tissue samples from patients	——	Exerted anti-cancer activities by aggravating mitochondrial impairment and ER stress through EphB4/CDC42/N-WASP signaling.	(19)
Lung cancer	A549 and NCI−H446	Human lung tumors xenograft model	Enhanced the effect of cisplatin by inhibiting the miR−23a/Akt/GSK3β signaling pathway and suppressing autophagy.	(145)
Cholangiocarcinoma	QBC and RBE	Tumor Xenograft model	Significantly lowered the expression of apoptosis‐related proteins (Bcl‐2, Bcl‐xL, and procaspase‐3), but increased the Bax expression. Resulted in the G0/G1 cell‐cycle arrest, reducing the expression of cell cycle‐related proteins (cyclin E, CDK2, and CDK4), and increased the expression of autophagy‐related proteins (LC3).	(42)
HCC	HepG2	Orthotopic HCC patient derived xenograft model	Disrupted of the HSP90 and CDC37 interaction and inhibited Raf/MEK/ERK pathway and PI3K/AKT/mTOR pathway.	(117)
		——	Induced HepG2 cells apoptosis through ROS-mediated mitochondrial dysfunction.	(51)
Pancreatic cancer	AsPC-1, BxPC-3, and PANC-1	——	Synergized with gemcitabine through abrogating Chk1/53BP1-mediated DNA repair.	(33)
			Caused G1 Arrest, induces apoptosis, and enhances the chemosensitivity to Gemcitabine. Inhibited translocation and DNA-binding activity of NF-kB.	(41)
	MiaPaCa-2 and Panc-1(PDA)	——	Inhibited hTERT expression by suppressing the transcription factors Sp1, c-Myc and NF-κB which control hTERT gene expression. PM also inhibited protein kinase Akt, which phosphorylates and facilitates hTERT nuclear importation and its telomerase activity.	(26)
			Induced apoptosis through the inhibition of pro-survival Akt/NF-κB/mTOR signaling proteins and anti-apoptotic Bcl-2.	(60)
Cervical Cancer	HeLa, CasKi, and SiHa	——	Induced Mitochondrial Cell Death by ROS-dependent activation of Bax and Poly (ADP-ribose) Polymerase-1	(56)
	Hela	Tumor xenografts on nude mice	Synergized with taxol to induce cell death by increasing intracellular ROS levels, upregulation of DR5, activation of Bax, and dissipation of mitochondrial membrane potential.	(130)
Ovarian carcinoma	OVCAR-5, MDAH- 2774, OVCAR-3, and SK-OV-3	——	Inhibited AKT/NF-k B/mTOR signaling pathway. Inhibited the expression of NF-κB-regulated antiapoptotic Bcl-2, Bcl-xL, C-IAP1 and survivin.	(57)
Osteosarcoma	MNNG (CRL1547) and 143B (CRL1427)	Human osteosarcoma xenograft model	Downregulated the levels of Akt, mTOR, and NF-κB.	(70)
Myeloma	H929 and U266	Human myeloma xenograft model	Inhibited proteasome chymotrypsin-like activity and NF-κB	(76)
OSCC	CAL-27 and SCC-25	——	Induced apoptosis *via* G1 phase arrest and MAPK/Erk1/2 and Akt signaling inhibition	(27)
Fibrosarcoma	HT1080	Mice with subcutaneous grafts comprising human fibrosarcoma cells	Inhibited cell and tumor proliferation by inhibiting AKT and MAPK signaling	(66)

CML, Chronic myeloid leukemia; CRC, colorectal cancer; CRLCs, conditionally reprogrammed patient-derived lung adenocarcinoma cells; ESCC, esophageal squamous cell carcinoma; HCC, hepatocellular carcinoma; NSCLC, non-small cell lung cancer; OSCC, oral squamous cell carcinoma; PDA, pancreatic ductal adenocarcinoma.

## Conclusion and Perspective

**Figure 3:**
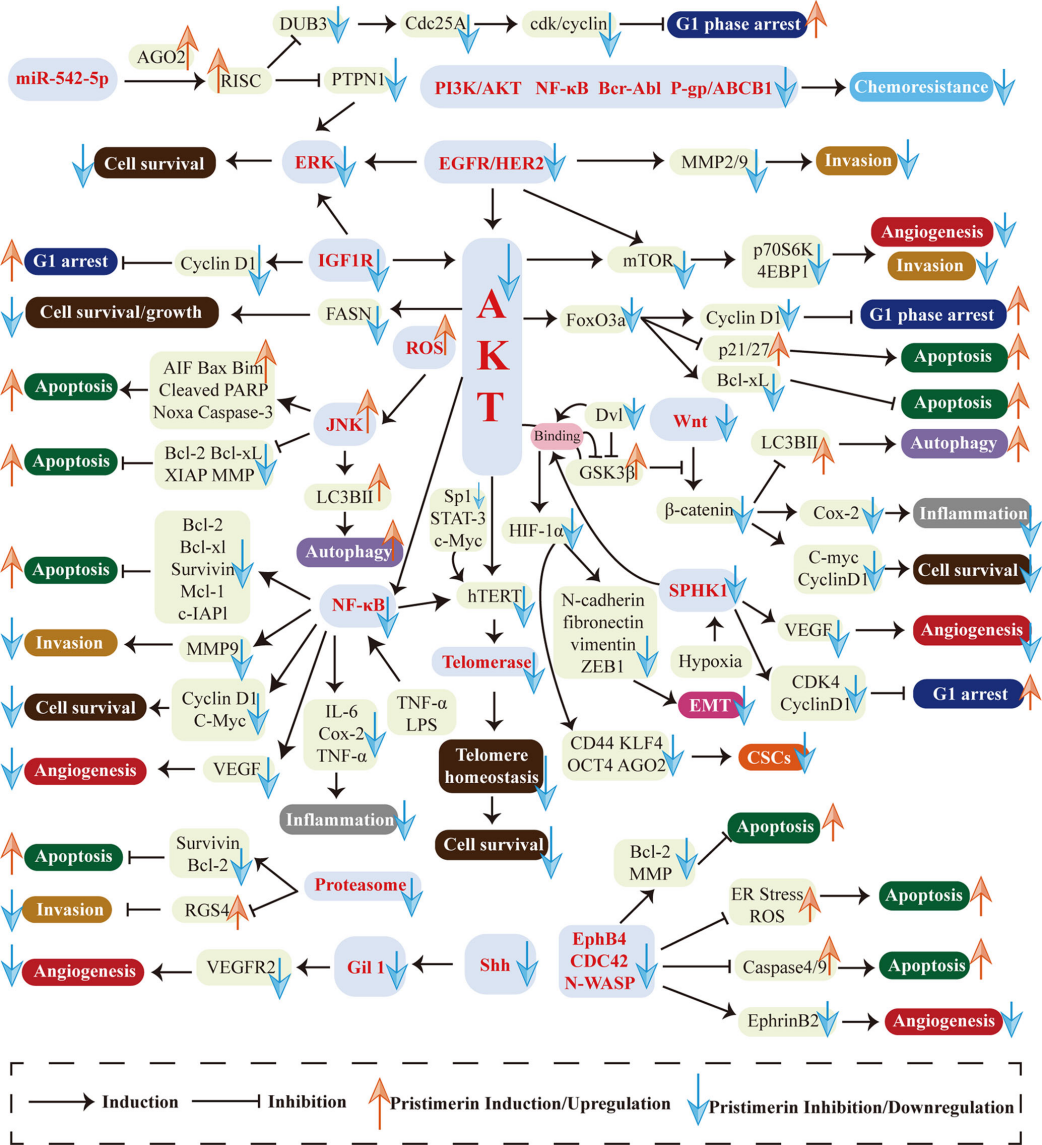
Multi-targeted molecular and biological mechanisms in pristimerin treatment. Various targets and pathways are involved in pristimerin treatment, including EGFR/HER2, IGF-1R, PI3K/AKT pathway, NF-κB pathway, ROS generation, MAPK pathway, EphB4/CDC42/N-WASP pathway, Wnt/β-catenin pathway, Shh/Gil1 pathway, HIF-1α/SPHK-1 pathway, Micro RNA, proteasome, telomerase, etc. These molecular events ultimately determine tumor cell fates including cell cycle arrest, apoptosis, autophagy, migration, invasion, metastasis, EMT, CSCs, angiogenesis, etc.

Natural compounds and herbs are attracting increasing attention for cancer chemo-treatment. Pristimerin, extracted from the Celastraceae and Hippocrateaceae families, exhibits potent anti-cancer activities in various cancers. This review has demonstrated the mechanisms and actions of pristimerin *in vitro* and vivo. As shown in **Figure 3**, a wide range of pathways and targets are involved during pristimerin treatment, some of which interact with each other to form a signal system regulating tumor cell fates together.

The first research hotspot is multi-targeted molecular and biological mechanisms associated with pristimerin treatment in cancers. Various targets and pathways are involved in pristimerin treatment, including EGFR/HER2 (38), IGF-1R (23), PI3K/AKT pathway (105), NF-κB pathway (43), ROS generation (28), MAPK pathway (27), EphB4/CDC42/N-WASP pathway (19), Wnt/β-catenin pathway (22), Shh/Gil1 pathway (17), HIF-1α/SPHK-1 pathway (24), Micro RNA (9), proteasome (25), telomerase (26), etc. These molecular events ultimately determine tumor cell fates including cell cycle arrest, apoptosis, autophagy, migration, invasion, metastasis, EMT, CSCs, angiogenesis, etc. Among them, there are several newly investigated pathways only found in one type of cancer currently such as Shh/Gli1 pathway in NSCLC (17), IGF-1R pathway in UM (23), EphB4/CDC42/N-WASP pathway in CRLC (19), and SPHK-1 pathway in prostate cancer (24). They need to be studied further in different cell lines, rather than being limited to a particular type of cell line and the interaction with other signaling systems should be elucidated as well. Although researchers have studied myriads of targets and pathways, disputes and unclear details still exist. For instance, given the fact that miR-542-5p stimulates the formation of RISC and then exhibits RNA silencing effects, two studies have reported different alterations of miR-542-5p expression and come to contrast conclusions about the effects of miR-542-5p on cancer cell proliferation. Cheng et al. have found that miR-542-5p is upregulated upon exposure to pristimerin, which may inhibit breast cancer cell proliferation (9). The other study has shown that miR-542-5p is downregulated in pristimerin-treated glioma and concluded that miR-542-5p may facilitate cancer cell proliferation (123). The reason for this disagreement has yet to be clarified. Furthermore, it is well known that pristimerin exhibits anti-oxidant activities through ameliorating oxidative stress (147, 148), but it can also result in the generation of ROS, which seems to be contradictory. These puzzles have enlightened us that the molecular mechanisms of pristimerin are complex and further studies are warranted to clarify the specific targets and pathways of pristimerin in cancer treatment.

For another, Li et al. have evaluated the ADME (absorption, distribution, metabolism, and excretion) properties of pristimerin to confirm that pristimerin may be a promising drug for cancers (123). It has also been found that pristimerin can be used in combination with myriads of common chemotherapeutic drugs to improve the sensitivity of tumor cells to chemotherapy and reverse drug resistance. What’s even more surprising is that pristimerin succeeds in inhibiting the proliferation of CML cells bearing T315I-Bcr-Abl which cannot be cured by currently available tyrosine kinase inhibitors (43). Additionally, pristimerin exerts anti-tumor effects in large quantities of cancers, especially in prostate cancer, breast cancer and CRC. Therefore, we can speculate that pristimerin will be a “treasure” for cancer patients in the future. However, there are few studies related to other common tumors, such as gastric cancer, renal cell carcinoma and lung cancer. Future researches are necessary to enrich the therapy spectrum of pristimerin.

Overall, the aim of pristimerin studies is to apply it into clinical use, improve the prognosis and diminish the tumor of patients. Considering its clinical application, the evaluation of adverse actions caused by pristimerin is essential. But the biggest obstacle is that there are no clinical trials on pristimerin, only experiments *in vitro* and in animals currently. Without clinical trials, we cannot make sure what kind of side effects will be caused when pristimerin is used in patients, and whether it will cause specific adverse reactions in humans when applied in combination with traditional chemotherapeutic drugs. Thus, pristimerin treatment is a promising new approach to eliminate tumors but further studies and clinical trials in humans are indispensable.

## Author Contributions

Z-GS and NZ designed the work. R-ZC wrote the manuscript. FY prepared the figures and tables. MZ drafted and revised the manuscript. All authors contributed to the article and approved the submitted version.

## Funding

This work was supported by the Shandong Provincial Natural Science Foundation (grant no. ZR2020MH204), the 19th batch of science and technology innovation development plan of Jinan in 2020 (Clinical medicine science and technology innovation plan, grant no.202019032), and the second group of science and technology projects of Jinan Health Committee (grant no. 2020-3-15).

## Conflict of Interest

The authors declare that the research was conducted in the absence of any commercial or financial relationships that could be construed as a potential conflict of interest.

## Glossary

**Table:**

ABC	ATP-binding cassette
AGO2	Argonaute 2
AIF	apoptosis-inducing factor
ALDH	aldehyde dehydrogenase
ASK1	apoptosis signal-regulating kinase 1
ATF6	activating transcription factor 6
BCRP	breast cancer resistance protein
CAC	colitis-associated colorectal cancer
CDKs	cyclin-dependent kinases
CDKIs	cyclin-dependent kinase inhibitors
CHOP	C/EBP homologous protein
CKI	cyclin-dependent kinase inhibitor
CML	chronic myelogenous leukemia
CRC	colorectal cancer
CRLCs	conditionally reprogrammed patient-derived lung adenocarcinoma cells
CSCs	cancer stem cells
Dvl	Dishevelled
EMT	epithelial-mesenchymal transition
EphB4	erythropoietin-producing hepatoma receptor B4
ERK	extracellular signal-regulated kinases
ESCC	esophageal squamous cell carcinoma
FASN	fatty acid synthase
Gli1	glioma-associated oncogene homolog 1
SK3β	glycogen synthase kinase-3 beta
HCC	hepatocellular carcinoma
HER2	human epidermal growth factor receptor 2
HIF-1α	hypoxia inducible factor 1α
hTERT	human telomerase reverse transcriptase
HUVECs	human umbilical vascular endothelial cells
IAPs	inhibitor of apoptosis proteins
IGF	insulin-like growth factor
IRE1α	inositol requiring kinase1 alpha
JNK	c-Jun NH2-terminal kinase or stress-activated protein kinases
MAPK	mitogen-activated protein kinase
MDR	multidrug resistance
MiRNA	micro RNA
MMPs	matrix metalloproteinases
MRP1	multidrug resistance-associated protein 1
mTOR	mechanistic target of rapamycin
NF-κB	nuclear factor-kappaB
NSCLC	non-small cell lung cancer
N-WASP	neural Wiskott-Aldrich syndrome protein
OSCC	oral squamous cell carcinoma
PARP	cleavage of poly (ADP-ribose) polymerase
PDA	pancreatic ductal adenocarcinoma
PERK	protein kinase-like ER kinase
P-gp	P-glycoprotein
PGPH	peptidyl-glutamyl peptide-hydrolyzing
PI3K	phosphatidylinositol 3-kinase
PTCH	Patched
PTPN1	protein tyrosine phosphatase nonreceptor type 1
Rb	retinoblastoma
RGS4	G protein signaling 4
ISC	RNA-induced silencing complex
ROS	reactive oxygen species
Shh	sonic hedgehog
SMO	Smoothened
SPHK1	sphingosine kinase 1
TCM	traditional Chinese medicine
TNFα	tumor necrosis factor-alpha
TNFR1	type 1 TNF receptor
TRAF2	TNFR-associated factor 2
UM	uveal melanoma
UPR	unfolded protein response
VEGF	vascular endothelial growth factor
